# Nutrition status of nulliparous married Indian women 15-24 years: Decadal trends, predictors and program implications

**DOI:** 10.1371/journal.pone.0221125

**Published:** 2019-08-27

**Authors:** Vani Sethi, Konsam Dinachandra, Zivai Murira, Jewel Gausman, Arti Bhanot, Arjan de Wagt, Sayeed Unisa, Salima Bhatia, Dinesh Baswal, S. V. Subramanian

**Affiliations:** 1 Nutrition Section, UNICEF India, Country Office, New Delhi, India; 2 International Institute for Population Sciences, Mumbai, India; 3 Regional Office for South Asia, UNICEF, Kathmandu, Nepal; 4 Women & Health Initiative, Department of Global Health and Population, Harvard T.H. Chan School of Public Health, Boston, MA, United States of America; 5 Independent Consultant, New Delhi, India; 6 Ministry of Health and Family Welfare, Government of India, Guwahati, Assam, India; 7 Harvard T.H. Chan School of Public Health, Boston, MA, United States of America; Burnet Institute, AUSTRALIA

## Abstract

In India, 66% of 8 million married adolescents (~5.3 million) are nulliparous and likely to conceive soon. Among married young women aged 20–24 years about 9.1 million are nulliparous. This group remains relatively less reached in maternal nutrition programs. Current estimates of their nutritional status and predictors of body mass index (BMI) are unavailable. Thinness (BMI <18.5 kg/m^2^), severe thinness (BMI <16 kg/m^2^), overweight or obesity (BMI **≥** 23kg/m^2^) prevalence estimates are presented based on a sample of 11,265 married nulliparous adolescents (15–19 years, married, no parity) and 15,358 young women (20–24 years, married, no parity) drawn from the National Family Health Surveys 2005–06 and 2015–16. Trends by age, time and state were analysed. Predictors of BMI were investigated using linear regression. Using BMI for age z score (BAZ) as standard reference, BMI cut-off was calculated for thinness (-2SD) and overweight or obesity (+1SD) among married nulliparous adolescents as recommended for population under 19 years. 35% sampled adolescents and 26% young women were thin; 4%-5% severely thin. Overweight or obesity was higher among married nulliparous young women than married nulliparous adolescents (21% versus 11%). Eight in 1000 were short, thin and young and six in 1000 were short, thin, anemic and young. At 15 years of age, prevalence of thinness based on BMI was 46.5% while based on BAZ, 7.6%. At 24 years of age thinness was 22.5%. Decadal reduction in thinness was half among married nulliparous adolescents (4% points) compared with married nulliparous young women (8% points). Decadal increase in overweight/ obesity ranged from 4% to 5% in both age groups. Western states had high prevalence of thinness; Tamil Nadu had highest prevalence of overweight or obesity. Incremental increase in age and wealth increased BMI among young women more than adolescents. BMI was lower among adolescents and young women wanting a child later than soon [β -0.28 (CI -0.49- -0.07), β -0.33(CI -0.56- -0.093), respectively]. BMI cut-off 16.49 kg/m^2^ and 24.12 kg/m^2^ had a high sensitivity (100%, 99.7%) and specificity (98.9%, 98.5%) to screen thin and overweight or obese adolescents, respectively. Owing to the high prevalence of both thinness and overweight/obesity among nulliparous married adolescents and women, nutritional anthropometry based screening should be initiated for this target group, along with a treatment package in states with high and persistent malnutrition. Family planning services should be integrated in nutrition programs for this target group to achieve normal nutritional status before conception.

## Introduction

Poor nutrition of women before and during pregnancy has profound effect on fetal growth and development as well as mother’s own health and well-being [1]. Studies have shown that children born to mothers who are short, young, thin or anaemic are more likely to experience in-utero growth restriction, born preterm or with low birth weight and be stunted; these mothers are at greater risk of dystocia (difficult labour) and haemorrhage [1,2,3,4,5,6,7,8]. Obese women have a higher risk of preeclampsia and gestational diabetes [9].

India witnesses 30 million pregnancies each year [10]. Most Indian women enter pregnancy with poor nutrition. Atleast 20% of women are thin (body mass index, BMI <18.5) and an equal proportion overweight (BMI ≥ 23), 53% anemic and 8% enter pregnancy as adolescents (~4.5 million) [11]. In the South Asian context, early marriage is strongly associated with early child bearing, lower likelihood of availing antenatal care services and poor maternal and newborn outcomes [12,13]. In India, the median age at marriage is 18.6 years and median age at first birth is 21 years [11]. A little over a quarter of adolescents aged 15–19 use modern contraceptives which further dips to less than 20% in 20–24 years age group. Research has shown that one-third of fertile couples not using contraception and having regular sex conceive within a month [14]. Among married women in India, about 66% of 8 million (~5.3 million) aged 15 to 19 years and 26% of 35 million (~9.1 million) aged 20–24 years are nulliparous, presenting an opportunity to meet any health and nutrition gaps before conception if family planning services are used [11, 15]. Delaying age at first birth is likely to improve woman’s nutritional status [16].

Formation of most fetal organs is initiated soon after conception and much before the woman arrives for the first antenatal check-up [7]. Studies conducted in several countries have shown that preconception energy and micronutrient supplementation increased birth weight while, interventions initiated after conception like improving pregnancy diet and micronutrient supplementation may improve women’s nutritional status but have limited impact on newborn health outcomes [17,18,19,20,21]. Excessive caffeine intake, alcohol consumption and smoking in preconception phase are associated with adverse fetal and newborn outcomes [22]. Hence, improving nutrition before pregnancy is critical.

However, women’s nutrition programs in India have traditionally focused on delivering nutrition services to women ‘during pregnancy’ through antenatal care delivery platforms. Antenatal services include basic health care to screen for and prevent pregnancy complications (history taking, clinical examination, two dose tetanus toxoid, laboratory investigations including urine test to confirm pregnancy, assess presence of sugars and proteins, blood investigations for haemoglobin estimation and blood grouping, including Rh factor, and rapid test for syphilis and malaria) and nutrition interventions (iron folic acid (IFA) and calcium supplementation, deworming and supplementary nutrition meeting a third of the days energy-protein needs) [23,24,25,26]. Provision of a basket of nutrition services to women before they enter pregnancy has been a challenge owing to huge numbers and no robust delivery platform among other factors. In high fertility districts, married women are targeted for family planning services albeit with limited reach, but no nutrition services are provided. Unmarried adolescents are covered under weekly IFA supplementation program, but once married often times they are also left out of both family planning and IFA supplementation programs.

There are not many small scale studies on the nutritional status of nulliparous women (15 to 24 years) in India [27]. Using larger data sets of National Family Health Survey (NFHS) 2005–06, it has been established that the prevalence of thinness is higher among pre-pregnant women than the average Indian women in the 15 to 49 years age group and women married before 18 years of age are thinner than those married after [28,29]. It is also known that drivers of malnutrition vary over time and should be ascertained periodically [30]. With the NFHS 2015–16 data sets now available, this paper presents 1) prevalence of thinness, severe thinness and overweight or obesity among married nulliparous adolescents (15–19 years) and married nulliparous young women (20–24 years), 2) determine proportion of most at risk married adolescents (MARA) or those short and thin, with or without anemia, 3) age, time and state-wise trends in prevalence of thinness and overweight or obesity and 4) predictors of both body mass index (BMI) and BMI for age z scores (BAZ) for adolescents and of BMI for young women. Finally, to address gaps in consensus on BMI cut-offs for adolescents, it aims to arrive at valid BMI cut-offs for screening thin and overweight or obese married adolescents using BAZ as standard reference.

## Methodology

### Sample

**Fig 1:**
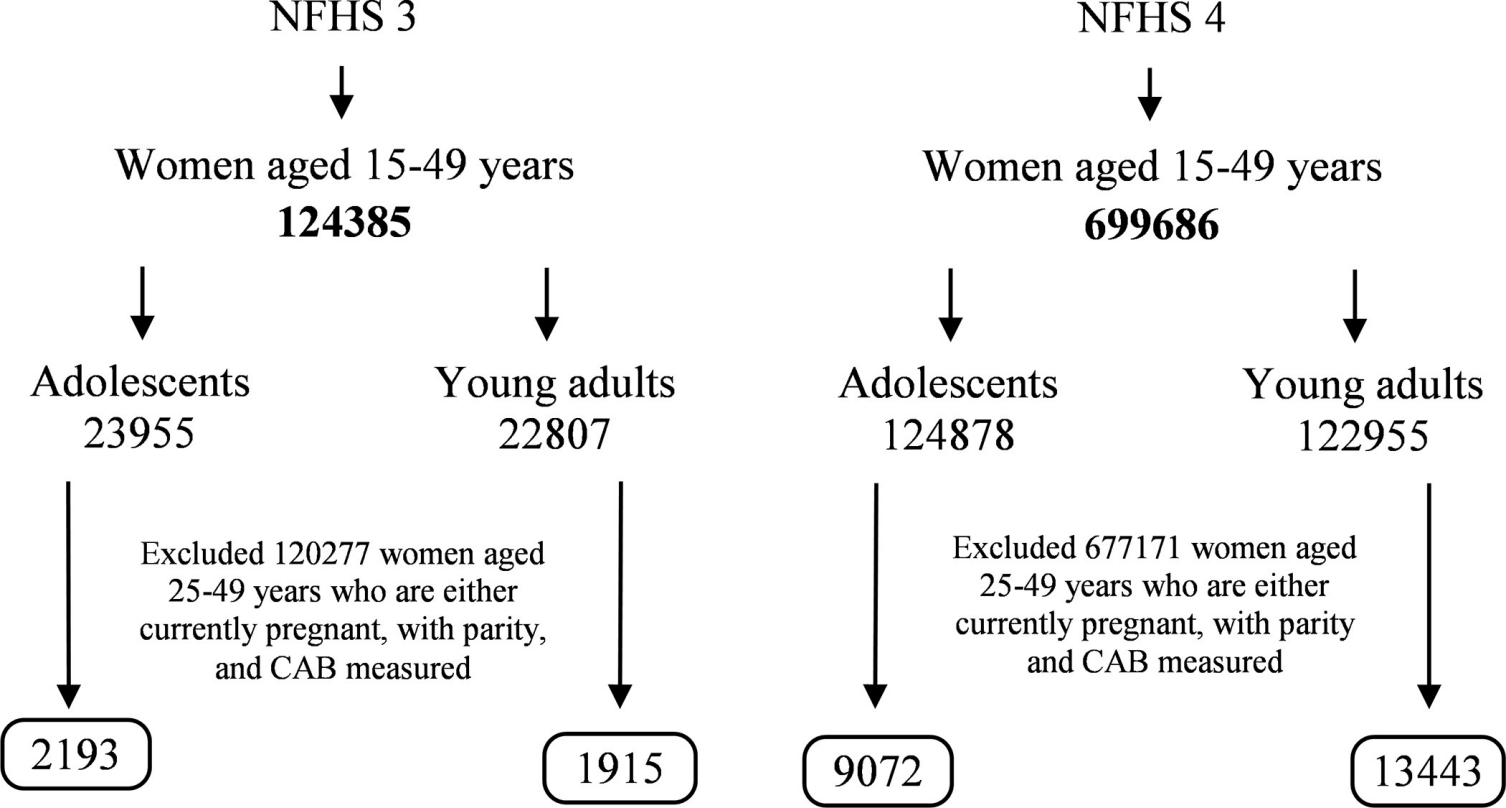
Analytical sample and sample size used in the study.

Data from two rounds of NFHS (also referred to as Demographic Health Survey), conducted during 2005–06 (NFHS-3) and 2015–16 (NFHS-4) were used in this study [11,31]. NFHS used two-staged, stratified cluster sampling design in which women of reproductive age were selected from a random sample of about 5,76,318 households. Starting with 1,24,385 women aged 15–49 years in NFHS 3 and 6,99,686 in NFHS 4, two age categories of 15–19 years (adolescent) and 20–24 years (young women) were created (n = 46,762 in NFHS 3 and n = 2,47,833 in NFHS 4). In each age category, only married women, neither currently pregnant nor having any offspring were included to get a sample of women most likely to conceive for the first time. Women who refused to give Clinical, Anthropometric and Biochemical (CAB) data on key anthropometric variables (i.e. height and weight) and biochemical testing (hemoglobin estimation) were excluded from the study. Thus, a final sample of 11,265 married nulliparous adolescents (2193 in NFHS 3 and 9072 in NFHS 4) and 15358 married nulliparous young women (1,915 in NFHS 3 and 13,443 in NFHS 4) were included in the study (Fig 1). To understand regional variations, analysis was limited to the states with a sample size of at least 30 as recommended for such analysis, and the states for which data from both rounds of NFHS were available [32,33].

### Primary outcome indicators of nutritional status

Three primary indicators of nutritional status were considered 1) thinness, 2) severe thinness and 3) overweight or obesity. Thinness was defined as BMI <18.5 kg/m^2^ for young women and both BMI <18.5 kg/m^2^ and BAZ <-2 SD for adolescents [34,35]. Severe thinness was defined as BMI <16 kg/m^2^ for both age group and additionally BAZ <-3 SD for adolescents. An Asian-specific BMI cut-off of **≥**23 kg/m^2^ was used to estimate overweight or obesity for both age groups; BAZ > +1SD was also used for adolescents [34]. A new variable of Most At Risk Adolescent (MARA) was created to assess the co-burden of different forms of undernutrition in this age group, which were defined by two different combinations of nutritional deprivation. Type-1 deprivations included adolescents with BAZ<-2SD and with short stature (height <145 cms). Type-2 deprivations were an extension of type-1 deprivations including anemia (Hb<12 mg/dL) [36,37].

### Independent variables

Based on desk review and theoretical relevance to preconception nutrition, independent variables included in the analysis were: age in individual years for both age groups; place of residence (rural and urban); education (no schooling, less than 5 years, 5–7 years, 8–9 years, 10–11 years and 12+ years); caste (scheduled caste (SC), scheduled tribe (ST), other backward class (OBC) and other, of which all except others are considered socially vulnerable); religion (Hindu, Muslim and other), state of residence (29 states and 7 union territories); fertility intention (desire for a child/another child soon or later, that is after two years or want no more) and wealth index based on scores on possession of pre-defined list of assets and five equal divisions of the distribution (poorest, poor, middle, rich and richest quintile) [3,6].

### Data analysis

To ensure representativeness of the results, national level sampling weights were used in the analysis. Chi square test was conducted to understand sample distribution based on parity by independent variables (individual and households characteristics). Descriptive univariate analysis of BMI, BAZ and MARA, was conducted to determine current prevalence of malnutrition in the study sample. Bi-variate analysis was conducted to estimate the age-specific, decadal and state-wise changes in prevalence of thinness, severe thinness and overweight.

Multiple linear regression analyses were used to examine the association between BAZ as well as BMI in married adolescents and BMI in married young women with selected individual and household characteristics using the formula $\hat{Y}=\beta_{0}+\beta_{1}X_{1}+\beta_{2}X_{2}+\cdots +\beta_{p}X_{p}$ where $\hat{Y}$ is the expected value of BAZ or BMI, *X*_1_ *through X_p_* are p distinct independent variables or predictors, *β*_0_ is the value of Y when the value of all predictors equals to 0 and *β*_1_ through *β_p_* are the estimated regression coefficients. In the age group of 15–19 years as both BAZ and BMI were considered for regression, four models were created (Model 1 and 2 without and with controlling other variables for BAZ and Model 3 and 4, without and with controlling other variables for BMI, respectively). Two regression models were specified for 20–24 years age group (Model 5 and 6). In Model 5, regression coefficients were presented for each variable without controls while in Model 6, adjustments for the effects of confounding variables were considered [38]. Statistical significance was defined as p value < 0.05. The Youden Index is a summary measure of the Receiver Operating Characteristic (ROC) curve, was used to arrive at realistic cut-offs for estimating undernutrition and obesity among married adolescent sample using BAZ <-2SD as standard for thinness and BAZ >+1 SD as standard for overweight or obese [39]. STATA v14 and MedCalc, version 18.11.3 were used for statistical analysis.

## Results

### Background characteristics of married nulliparous adolescents and married nulliparous young women

Table 1 provides the background characteristics of the sampled adolescents and young women. Majority of the women in both age groups were rural residents (82% among 15–19 years, 69% among 20–24 years) and practicing Hinduism (over 80% on both groups). More than half of the women in both age-groups belonged to socially vulnerable groups. Among adolescents, higher proportion belonged to lowest and second lowest quintile, while among young women higher proportion belonged in middle and higher wealth quintiles. Over a third of the sampled adolescents were under 18 years of age. While the proportion with no schooling was under 15% in both age-groups, 38% had 12 years or more of education among married nulliparous young women opposed to 15% among married nulliparous adolescents. Among married nulliparous adolescents 58% wanted a child soon and this increased to over 70% among young women

**Table 1:** Background characteristics of married nulliparous adolescents (15–19 years) and married nulliparous young women (20–24 years) (NFHS-4).

	Adolescents (15–19 years) (N = 9072)		Young women (20–24 years) (N = 13443)	
	n	%	n	%
Residence				
Urban	1363	17.7	3457	31.0
Rural	7709	82.3	9986	69.0
Age of women				
15/20	474	5.5	3872	28.1
16/21	864	10.0	2550	19.1
17/22	1396	15.6	2942	22.2
18/23	3368	36.7	2228	17.2
19/24	2970	32.3	1851	13.5
Schooling				
No schooling	1479	14.9	2129	14.4
Completed <5 years	425	4.7	477	3.5
Completed 5–7 years	1572	17.0	1772	13.5
Completed 8–9 years	2660	28.3	2533	16.6
Completed 10–11 years	1745	20.6	1849	14.1
12 or more years complete	1191	14.6	4683	37.9
Desire for children				
Want soon	5148	58.2	9582	72.0
Want later	2862	30.6	2573	18.9
Want no more	296	3.3	293	2.1
Uncertain	766	7.9	995	7.0
Religion				
Hindu	7521	83.9	10821	82.7
Muslim	1111	13.4	1657	13.5
Other	440	2.8	965	3.8
Caste/tribe				
Scheduled caste	1935	22.7	2490	20.3
Scheduled tribe	1353	11.4	2046	9.0
Other backward class	4208	45.8	5929	45.9
Other	1576	20.1	2978	24.8
Wealth index				
Lowest	2579	26.1	2416	16.0
Second	2648	28.1	2812	19.5
Middle	1957	21.9	2879	21.5
Fourth	1276	16.3	2736	22.2
Highest	612	7.6	2600	20.9
Total (N)	9072		13443	

### Prevalence of thinness, severe thinness and overweight or obesity

Based on most recent estimates from NFHS 4, among married nulliparous adolescents, 35% were thin and 5% were severely thin, while among young women 26.3% were thin and 4.4% severely thin. Between the two rounds of survey, the proportion thin and severely thin declined in both age groups; by 3.9% points among married nulliparous adolescents while by 8.2% points among young women. Prevalence of overweight or obesity was 10.9% among married nulliparous adolescents, compared with 21.4% among young women. Prevalence of overweight or obesity increased in both age groups; by 4.3% points among married nulliparous adolescents and 5.3% points among young women. Expectedly, mean BMI was higher for young women at 20.7, compared with 19.7 among adolescents (Table 2).

**Table 2:** Mean BMI, severe thinness (BMI<16 kg/m^2^), thinness (BMI<18.5 kg/m^2^) and overweight or obesity (BMI ≥23 kg/m^2^) among married nulliparous adolescents (15–19 years) and married nulliparous young women (20–24 years), NFHS 3 and NFHS 4.

				Body Mass Index (kg/m^2^)		
	Age group	N	Mean BMI	<16	<18.5	≥23
				% (n)	% (n)	% (n)
NFHS 4	15–19	9072	19.7	5.0 (455)	35.0 (3178)	10.9 (990)
	20–24	13443	20.7	4.4 (596)	26.3 (3537)	21.4 (2875)
NFHS 3	15–19	2193	19.4	6.3 (138)	38.9 (852)	6.6 (145)
	20–24	1915	20.3	6.0 (115)	34.5 (660)	16.1 (309)

### Proportion of Most At Risk Adolescent (MARA)

**Fig 2:**
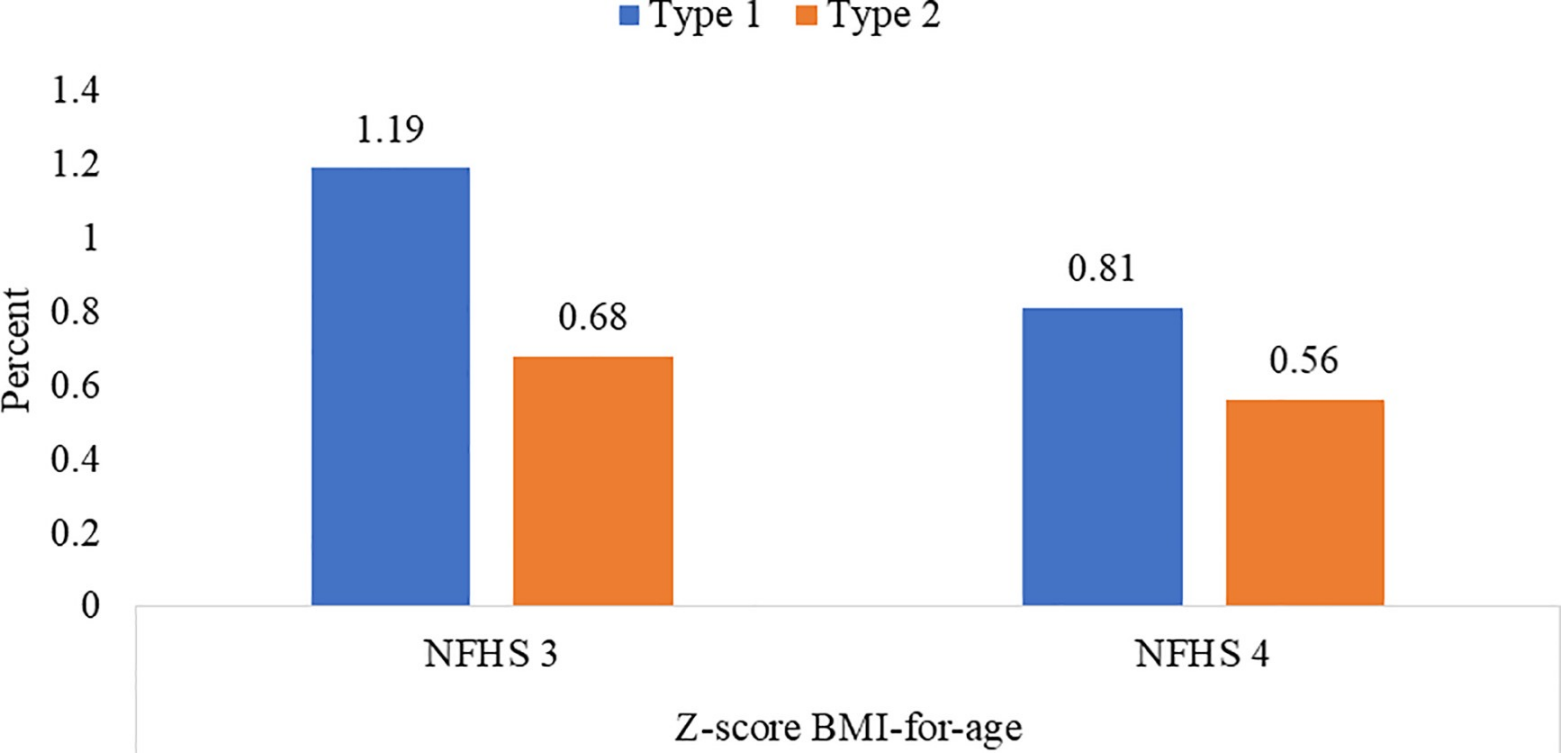
MARA by type 1 and type 2* (15–19 years) (%), NFHS -3 and NFHS -4. * Type– 1 deprivations = BAZ <- 2SD, height <145 cms, Type– 2 deprivation = Type -1 deprivation and anemia (Hb <12 g/ dL).

Married nulliparous adolescents have increased vulnerability due to risk of early pregnancy. Eight in 1000 suffered from co-burden of both short stature and thinness, while almost six in 1000 suffered from triple burden of short stature, thinness and anemia (Fig 2).

### Age variations

**Fig 3:**
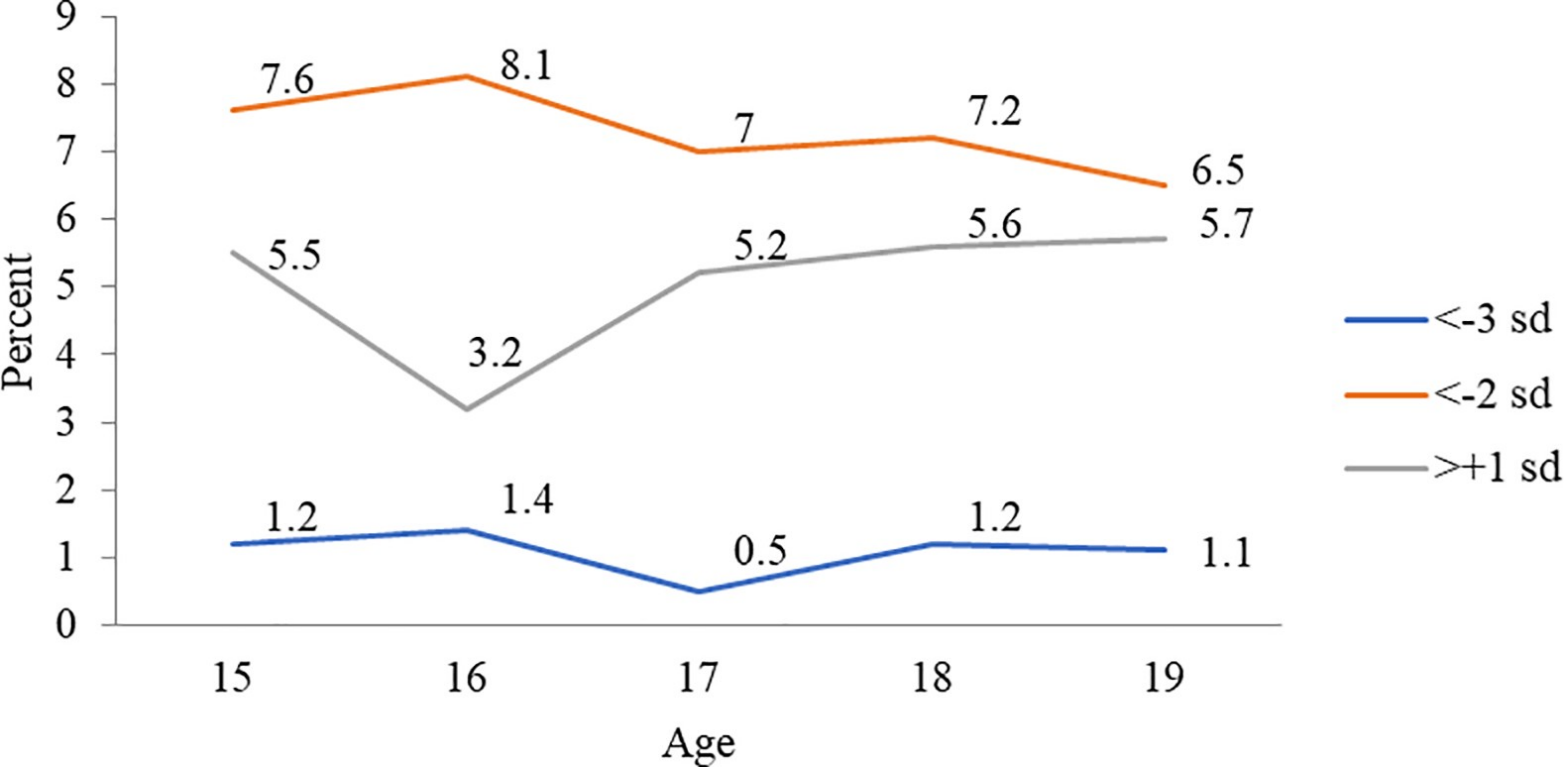
Thinness (BAZ <-2SD), severe thinness (BAZ <-3SD) and overweight or obesity (BAZ >+1SD) among married nulliparous adolescents (15–19 years) (%) by age, NFHS-4.

**Fig 4:**
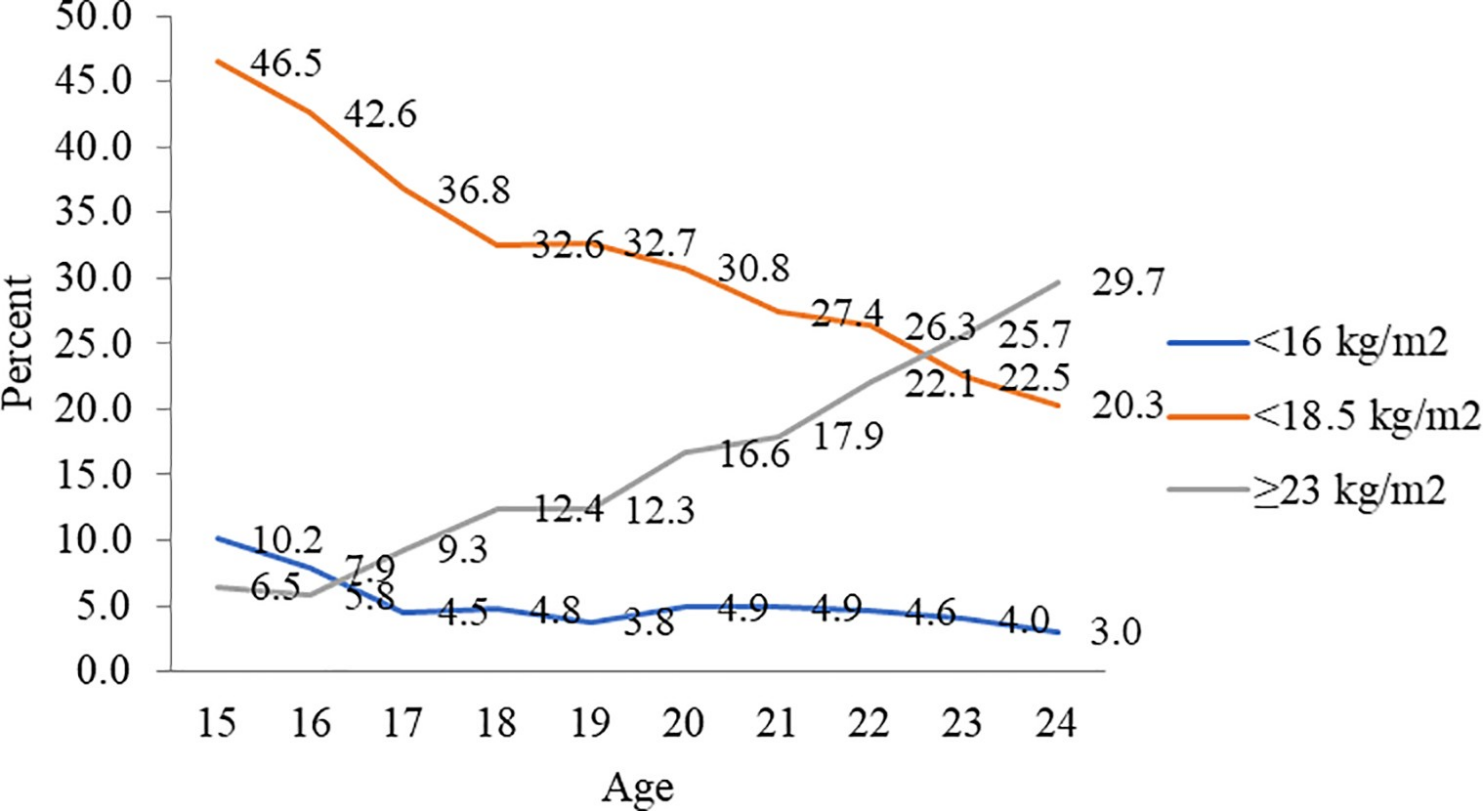
Thinness (BMI<18.5 kg/m2), severe thinness (BMI<16 kg/m2), and overweight or obesity (BMI ≥ 23 kg/m2) among married nulliparous adolescents and young women (15–24 years) (%) by age, NFHS-4.

Prevalence estimates for thinness, severe thinness and overweight or obesity among married nulliparous adolescents based on estimates using BAZ were much lower than those obtained using standard BMI cut-offs. Based on BAZ estimates, prevalence of thinness was higher among women/girls aged 15 and 16 years than 17 or older. The severity of thinness peaked at 16 years at 1.4%. Prevalence of overweight or obesity was over 5% for all ages except at 16 years (Fig 3). However, using BMI cut-offs, a steep decline in prevalence of thinness was observed from ages 15 years (46.4%) to 18 years (32.5%). At a prevalence of 10%, severity of thinness was a concern for 15 years aged married nulliparous adolescents. Prevalence of obesity was higher at 18 and 19 years at over 12% (Fig 4). Among 20–24 years aged married women, the prevalence of thinness and severe thinness decreased with each completed year from 30.8% at 20 years to 20.3% at 24 years. Concomitantly, prevalence of overweight or obesity increased from 16.6% at 20 years to 29.7% at 24 years (Fig 4).

### Decadal trends

**Fig 5:**
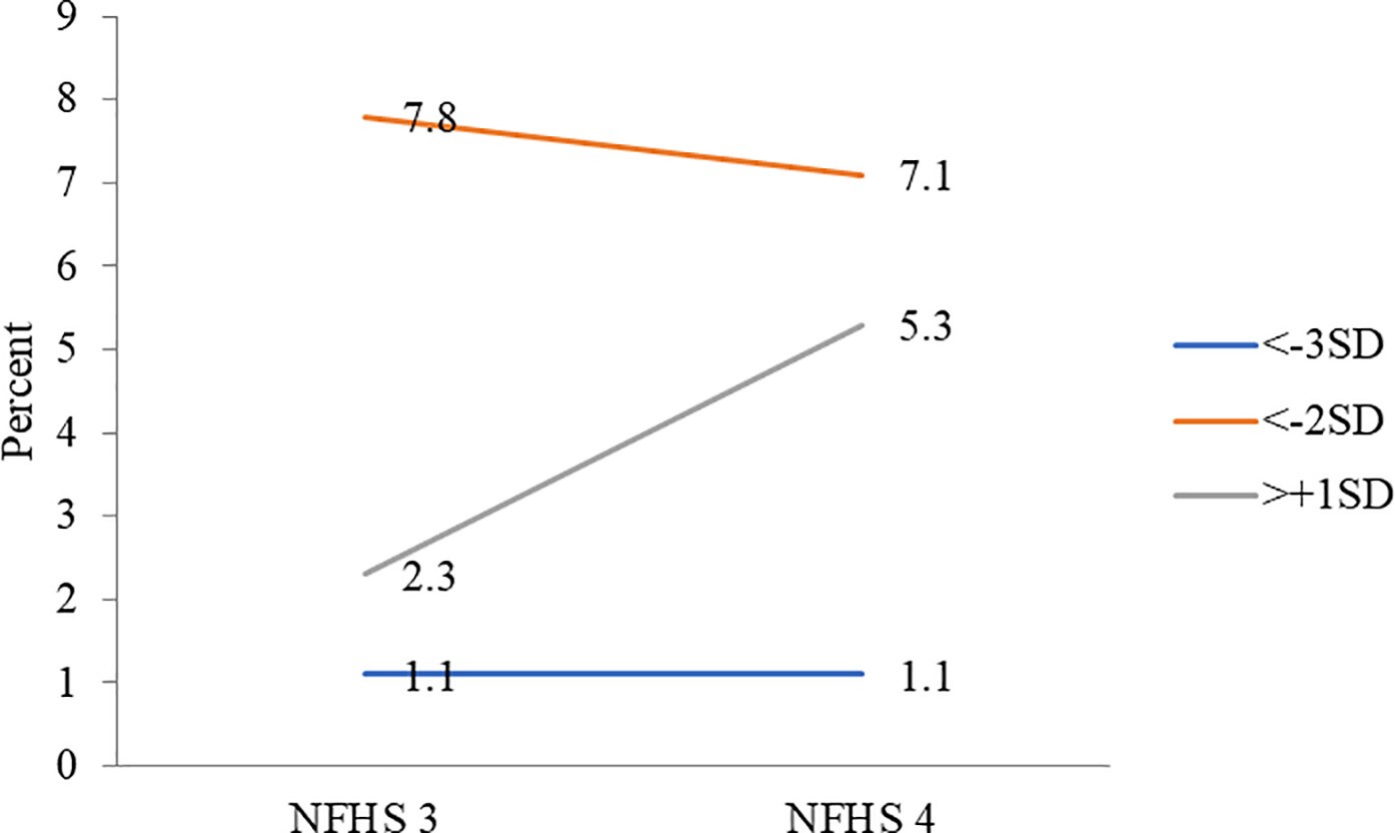
Thinness (BAZ <-2SD), severe thinness (BAZ <-3SD) and overweight or obesity (BAZ >+1SD) among married adolescents (15–19 years) (%), NFHS-3 and NFHS-4.

The prevalence of thinness declined and that of overweight or obesity increased across both age groups. Based on BAZ, prevalence of thinness reduced marginally from 7.8% to 7.1% among 15–19 years aged married nulliparous adolescents while that of severe thinness remained unchanged at 1.1%. Prevalence of overweight/ obesity increased from 2.3% to 5.3% (Fig 5). Among 20–24 years aged married nulliparous women, prevalence of both thinness and severe thinness declined (34.5% to 26.3% and 6% to 4.4%, respectively) and prevalence of overweight/ obesity increased from 16.1% to 21.4% (Table 2).

### Regional variations

#### Married nulliparous adolescents (15–19 years)

Table 3 provides estimates of severe thinness, thinness, and overweight among married nulliparous adolescents in selected states by region in India using NFHS 3 and 4 data. Based on BAZ, the highest prevalence of thinness and severe thinness was observed in Gujarat (16% and 3.7%, respectively) and lowest in Arunachal Pradesh (2% and 0, respectively). Highest prevalence of overweight or obesity was noted in Tamil Nadu at 16.9%. When BMI cut-offs were applied, Gujarat continued to have the highest prevalence of both thinness and severe thinness at 50% and 12% respectively. Similarly, Tamil Nadu had the highest prevalence of overweight/ obesity at 33%. Based on BMI cut-off, four states had a thinness prevalence of over 40%- Gujarat 49.6%, Rajasthan 44.1%, Karnataka 43.2% and Maharashtra 41.4%, five states had a prevalence from 30% to 40%- Madhya Pradesh 37.5%, Chhattisgarh 36.3%, Bihar 35.5%, Jharkhand 34.3% and West Bengal 30.4% and six had between 20% to 30%- Uttar Pradesh 29.8%, Punjab 29.1%, Andhra Pradesh 27.5%, Odisha 26.4%, Assam 26% and Haryana 25.4%. The prevalence of thinness declined in13 of 17 states from 2005–06 (NFHS 3) to 2015–16 (NFHS 4), exceptions being the high prevalence states of Gujarat, Karnataka, Madhya Pradesh and Rajasthan. Further, severe form of thinness increased in Gujarat between the two rounds. Between the two rounds of NFHS survey, in addition to Tamil Nadu two states- Punjab and Arunachal Pradesh had prevalence of overweight or obesity higher than 20% and 11 of the 17 states had prevalence higher than 10%. Prevalence of overweight/ obesity increased across all states except Rajasthan.

**Table 3:** Prevalence of thinness (BAZ <-2SD or BMI < 18.5 kg/m^2^), severe thinness (BAZ <-3SD or BMI <16 kg/m^2^) and overweight or obesity (BAZ >+1SD or BMI ≥ 23 kg/m^2^) among married nulliparous adolescents (15–19 years) by selected Indian states (NFHS-3, NFHS-4).

	z-score of BMI-for-age						Body Mass Index (BMI in kg/m^2^)						Sample size	
	<-3SD (%)		<-2SD (%)		>+1SD (%)		<16 (%)		<18.5 (%)		≥23 (%)			
	NFHS 3	NFHS 4	NFHS 3	NFHS 4	NFHS 3	NFHS 4	NFHS 3	NFHS 4	NFHS 3	NFHS 4	NFHS 3	NFHS 4	NFHS 3	NFHS 4
INDIA	1.1	1.1	7.8	7.1	2.3	5.3	6.3	5.0	38.9	35.0	6.6	10.9	2193	9072
**North**														
Haryana	0.0	0.9	4.6	5.6	3.0	4.7	3.0	2.8	37.3	25.4	9.1	14.7	64	165
Punjab	0.0	0.0	5.5	7.1	2.7	11.4	5.5	5.0	35.5	29.1	8.0	20.5	37	73
Rajasthan	0.9	1.7	11.0	10.5	2.5	2.1	9.1	7.4	43.1	44.1	6.1	6.2	208	1135
**Central**														
Chhattisgarh	1.2	0.3	4.9	8.4	3.2	5.4	3.7	5.5	37.6	36.3	4.4	13.2	87	164
Madhya Pradesh	1.3	0.4	10.0	6.8	2.0	3.0	8.7	4.2	37.3	37.5	5.3	7.3	190	1028
Uttar Pradesh	0.8	0.7	6.2	5.3	1.1	4.4	5.0	3.3	34.5	29.8	7.0	9.8	368	1311
**East**														
Bihar	1.4	1.1	4.7	6.1	1.6	3.1	4.6	4.6	45.4	35.5	2.9	7.8	190	1405
Jharkhand	0.5	1.2	5.6	6.3	0.0	1.6	2.9	3.7	35.6	34.3	1.8	4.2	117	617
Odisha	0.0	0.5	10.6	5.3	3.8	8.2	6.0	4.3	32.4	26.4	12.8	14.6	73	329
West Bengal	2.6	0.3	10.4	6.0	2.8	6.6	6.9	3.8	39.7	30.4	8.8	15.6	139	343
**Northeast**														
Arunachal Pradesh	0.0	0.0	6.4	2.0	0.0	12.5	2.9	1.3	25.4	17.1	3.4	20.2	31	111
Assam	0.0	0.4	4.4	4.3	3.5	3.8	0.9	3.6	37.7	26.0	6.1	9.3	62	388
**West**														
Gujarat	1.5	3.7	12.1	16.0	1.5	5.6	9.1	12.3	46.9	49.6	9.1	10.1	66	356
Maharashtra	1.4	0.8	13.9	6.6	2.2	5.7	10.6	5.9	43.6	41.4	4.2	9.5	88	416
**South**														
Andhra Pradesh	2.2	0.0	6.9	2.2	5.1	13.3	6.6	1.1	30.6	27.5	11.0	18.4	117	134
Karnataka	0.0	3.1	7.3	11.8	4.4	7.5	7.3	7.7	40.4	43.2	9.5	11.4	97	287
Tamil Nadu	1.0	0.7	11.4	4.8	5.8	16.9	11.4	3.5	43.3	19.4	11.8	33.0	47	143

#### Young nulliparous young women (20–24 years)

Table 4 presents regional variation in the prevalence of severe thinness, thinness, and overweight or obesity among married women in the age group 20–24 years. Estimates were available from 16 states opposed to 17 states for the younger age group as Arunachal Pradesh was excluded due to sample size less than 30. Among 20–24 years age group, the highest prevalence of thinness and severe thinness was noted in Gujarat at 36.8% and 10.1%, respectively, and that of overweight or obesity in Tamil Nadu at 39.3%. The lowest prevalence of thinness was in Tamil Nadu (14.4%) and that of overweight/ obesity was in Jharkhand (13.1%). Between the two rounds of NFHS survey, prevalence of thinness declined in 13 of 16 states with exception of Gujarat, Rajasthan and Uttar Pradesh. As in younger age group, prevalence of severe thinness increased in Gujarat. Except for Rajasthan, prevalence of overweight/ obesity increased in all states but with considerable variations.

**Table 4:** Prevalence of severe thinness (BMI <16 kg/m^2^), thinness (BMI < 18.5 kg/m^2)^ and overweight or obesity (BMI > 23 kg/m^2^) among married nulliparous women (20–24 years) in selected Indian states (NFHS-3, NFHS-4).

	BMI (kg/m^2^)						Sample size	
	<16		<18.5		> = 23			
	NFHS 3	NFHS 4	NFHS 3	NFHS 4	NFHS 3	NFHS 4	NFHS 3	NFHS 4
	%	%	%	%	%	%	n	n
INDIA	6.03	4.43	34.47	26.31	16.13	21.39	1915	13443
**North**								
Haryana	2.4	3.7	30.0	20.0	12.8	20.6	47	483
Punjab	4.1	2.7	23.9	14.9	23.7	27.9	76	257
Rajasthan	5.6	5.7	34.1	33.3	13.0	15.2	114	1313
**Central**								
Chhattisgarh	9.0	4.2	43.2	34.5	6.6	14.4	57	439
Madhya Pradesh	10.5	4.1	49.5	28.1	8.7	13.9	148	1360
Uttar Pradesh	3.2	2.5	23.4	23.8	20.1	18.9	194	2431
**East**								
Bihar	4.4	4.8	38.5	29.8	13.7	14.5	67	1,019
Jharkhand	8.0	4.8	41.7	30.4	8.8	13.1	44	560
Odisha	3.9	3.5	40.3	25.8	9.7	20.7	86	587
West Bengal	3.4	3.2	34.7	21.9	17.5	32.5	91	283
**Northeast**								
Assam	4.7	3.1	33.4	28.4	23.7	19.5	40	420
**West**								
Gujarat	7.4	10.1	32.9	36.8	15.1	20.2	94	583
Maharashtra	11.4	5.8	48.2	25.3	10.1	23.4	119	584
**South**								
Andhra Pradesh	7.6	7.0	33.5	31.8	13.3	26.5	137	170
Karnataka	4.6	3.6	37.9	29.8	15.8	18.0	84	525
Tamil Nadu	7.5	2.8	30.4	14.4	37.1	39.3	100	380

### Predictors of BAZ and BMI among married nulliparous adolescents

Table 5 presents regression results examining associations between key demographic characteristics and BAZ and BMI among married nulliparous adolescents. In the unadjusted model (Models 1 and 3), married nulliparous adolescents in urban areas were likely to have a higher BAZ [β 0.22 (CI 0.11–0.33)] and BMI [β 0.77 (CI 0.43–1.1)] than their rural counterparts. This difference was not observed after adjustment (Model 2) with BAZ and the effect was reduced for BMI [β 0.31 (CI -0.05–0.67)] (Model 4). Age made no difference to BMI in adjusted model (Model 4), however women aged 19 years had lower BAZ than 15 year olds [β -0.14 (CI -0.29–0.01)] post adjustment (Model 2). According to the results of both models 1 and 2, any length of schooling was associated with significant increases in BAZ score. Maximum gain was observed among those schooled for at least 12 years [β 0.28 (CI 0.17–0.39), unadjusted model]. However, such association was not observed when BMI was considered. Post adjustment, only 12 or more years of education increased BMI [β 0.36 (CI -0.02–0.73)]. Those wanting children later, not wanting any more children or uncertain had lower BAZ and BMI than those wanting soon in both adjusted and unadjusted models. In the adjusted model, those belonging to ST and OBC were likely to have a lower BAZ and BMI than non-vulnerable ethnic groups. The effect was more significant for those belonging to tribal households [BAZ, β -0.17 (CI -0.28- -0.05) and BMI, β -0.46 (CI -0.8 - -0.13)]. Belonging to the fourth and fifth quintile (richer or richest) increased both BAZ and BMI while middle and lower quintile had no effect. Being in richest quintile increased BMI [β 0.92 (CI 0.4–1.4)] and BAZ [β 0.27 (CI 0.9–0.45)].

**Table 5:** Unadjusted and adjusted effect of BAZ and BMI among married nulliparous adolescents (15–19 years) by selected background characteristics in India, NFHS-4.

(N = 12992)	BAZ (SD)						BMI (kg/m2)					
	Model -1			Model -2			Model -3			Model -4		
	Coefficient (β)	95% CI		Coefficient(β)	95% CI		Coefficient(β)	95% CI		Coefficient(β)	95% CI	
		Lower	Upper		Lower	Upper		Lower	Upper		Lower	Upper
**Place of residence**												
Rural^R^												
Urban	0.22***	0.11	0.329	0.08	-0.029	0.198	0.77***	0.43	1.101	0.31*	-0.049	0.667
**Age of women**												
15 ^R^												
16	-0.11	-0.268	0.056	-0.12	-0.279	0.033	-0.19	-0.83	0.456	-0.24	-0.881	0.404
17	-0.04	-0.196	0.119	-0.09	-0.241	0.064	0.33	-0.324	0.98	0.18	-0.461	0.827
18	-0.02	-0.16	0.128	-0.09	-0.233	0.05	0.58*	-0.04	1.2	0.35	-0.267	0.976
19	-0.03	-0.172	0.119	-0.14*	-0.285	0.005	0.68**	0.054	1.301	0.33	-0.285	0.955
**Years of schooling**												
No Schooling ^R^												
<5 years complete	0.12	-0.024	0.257	0.1	-0.036	0.243	0.17	-0.231	0.579	0.13	-0.278	0.529
5–7 years complete	0.12**	0.025	0.22	0.09*	-0.007	0.193	0.22	-0.097	0.528	0.13	-0.194	0.447
8–9 years complete	0.13***	0.04	0.222	0.09*	-0.007	0.182	0.25	-0.06	0.557	0.13	-0.182	0.443
10–11 years complete	0.2***	0.112	0.295	0.13**	0.026	0.231	0.52***	0.218	0.828	0.24	-0.066	0.553
12 or more years complete	0.28***	0.168	0.397	0.17**	0.039	0.291	0.89***	0.514	1.26	0.36*	-0.023	0.734
**Desire for children**												
Want soon ^R^												
Want later	-0.1***	-0.163	-0.03	-0.11***	-0.176	-0.04	-0.35***	-0.561	-0.136	-0.28**	-0.488	-0.068
Want no more	-0.13*	-0.273	0.016	-0.13*	-0.27	0.016	-0.57***	-0.955	-0.184	-0.4**	-0.773	-0.019
Uncertain	-0.11*	-0.221	0	-0.12**	-0.23	-0.009	-0.41**	-0.743	-0.08	-0.35**	-0.677	-0.013
**Ethnicity**												
Others ^R^												
Schedule caste	-0.11**	-0.213	-0.01	-0.05	-0.152	0.053	-0.29*	-0.613	0.031	-0.1	-0.415	0.224
Schedule tribe	-0.24***	-0.362	-0.128	-0.17***	-0.285	-0.052	-0.71***	-1.047	-0.376	-0.46***	-0.8	-0.129
OBC	-0.13***	-0.216	-0.037	-0.09*	-0.177	0.001	-0.38***	-0.648	-0.122	-0.26*	-0.52	0.005
**Wealth Index**												
Poorest ^R^												
Poorer	0.01	-0.06	0.071	-0.03	-0.1	0.035	0.04	-0.169	0.241	-0.08	-0.269	0.115
Middle	0.09**	0.016	0.169	0.03	-0.05	0.115	0.35***	0.112	0.588	0.15	-0.091	0.391
Richer	0.27***	0.17	0.378	0.18***	0.073	0.289	0.92***	0.587	1.251	0.6***	0.263	0.93
Richest	0.4***	0.239	0.559	0.27***	0.094	0.451	1.41***	0.929	1.885	0.92***	0.402	1.446

Note:

^R^ Reference category

* p<0.10

** p<0.05 &

*** p<0.01

### Predictors of BMI among married young women

Table 6 presents regression results examining associations between key demographic characteristics and BMI among married nulliparous young women. Living in urban areas improved BMI even after controlling for other variables [β 0.68 (CI 0.42–0.95)]. Compared with 20 years aged women, 22, 23 and 24 years aged women had higher BMI [β 0.45 (CI 0.18–0.72), β 0.48 (CI 0.19–0.76) and β0.88 (CI 0.57–1.19), respectively]. Schooling of at least 12 years as well as 8–9 years improved BMI. Similar to findings in 15–19 years age-group, those wanting children later had lower BMI than those wanting soon. However, unlike adolescents, belonging to any vulnerable social group reduced BMI and belonging to any wealth quintile higher than the lowest, increased BMI. BMI among those belonging to richest quintile was higher compared with poorest [β 1.18 (CI 0.84–1.53)].

**Table 6:** Unadjusted and adjusted effect of BMI among young married nulliparous women (20–24 years) by selected background characteristics in India, NFHS-4.

	BMI (kg/m2)					
	Young married women (20–24 years)					
	N = 13443					
	Model -5			Model -6		
	Coefficient(β)	95% CI		Coefficient (β)	95% CI	
		Lower	Upper		Lower	Upper
Place of residence						
Rural ^R^						
Urban	1.27***	1.026	1.506	0.68***	0.415	0.952
Age of women						
20 ^R^						
21	0.17	-0.086	0.418	-0.03	-0.273	0.221
22	0.65***	0.372	0.925	0.45***	0.178	0.716
23	0.81***	0.523	1.106	0.48***	0.194	0.764
24	1.3***	0.979	1.615	0.88***	0.568	1.195
Years of schooling						
No Schooling ^R^						
<5 years complete	0.05	-0.423	0.521	-0.02	-0.476	0.431
5–7 years complete	0.25	-0.089	0.594	-0.06	-0.402	0.279
8–9 years complete	0.66***	0.355	0.97	0.37**	0.069	0.672
10–11 years complete	0.76***	0.457	1.056	0.21	-0.092	0.517
12 or more years complete	1.2***	0.945	1.457	0.33**	0.051	0.615
Desire for children						
Want soon ^R^						
Want later	-0.24*	-0.484	0.005	-0.33***	-0.56	-0.093
Want no more	-0.53	-1.259	0.208	-0.57	-1.265	0.123
Others	-0.24	-0.633	0.156	-0.29	-0.705	0.12
Ethnicity						
Others ^R^						
Schedule caste	-0.99***	-1.275	-0.699	-0.55***	-0.842	-0.261
Schedule tribe	-1.6***	-1.925	-1.271	-0.85***	-1.191	-0.506
OBC	-0.71***	-0.969	-0.442	-0.41***	-0.67	-0.148
Wealth Index						
Poorest ^R^						
Poorer	0.45***	0.206	0.702	0.29**	0.038	0.536
Middle	1.14***	0.858	1.419	0.72***	0.453	0.987
Richer	1.36***	1.088	1.628	0.72***	0.394	1.039
Richest	2.15***	1.864	2.429	1.18***	0.835	1.53

Note:

^R^ Reference category

* p<0.10

** p<0.05 &

*** p<0.01

### BMI cut-offs for adolescents

Using NFHS 4 data, the optimal BMI cut-off to detect thinness amongst married nulliparous adolescents was ≤16.49 kg/m^2^ and for overweight or obesity >24.12 kg/m^2^ (Table 7).

**Table 7:** Sensitivity, specificity, and Youden Index at cut-off values of BMI (kg/m^2^) for diagnosing thinness and overweight or obesity among married nulliparous adolescents (15–19 years), NFHS-4.

	BMI cut off	Sensitivity	Specificity	Youden Index	Area under curve	P-group	N
Thinness							
15	≤15.76	100	100	1	1	8.23	474
16	≤16.12	100	100	1	1	6.48	864
17	≤16.33	100	100	1	1	6.81	1396
18	≤16.44	100	100	1	1	7.21	3368
19	≤16.49	100	100	1	1	6.43	2970
15–19	≤16.49	100	98.9	0.99	1	6.88	9072
Overweight							
15	>23.40	100	100	1	1	3.16	474
16	>24.01	100	100	1	1	3.24	864
17	>24.51	100	100	1	1	3.51	1396
18	>24.75	100	100	1	1	4.51	3368
19	>24.98	100	100	1	1	4.75	2970
15–19	>24.12	99.74	98.5	0.98	1	4.24	9072

## Discussion

The analysis determines nutritional status of married nulliparous adolescents and married nulliparous young women who are most likely to conceive for the first time. Married nulliparous adolescents were found to have a lower mean BMI than married nulliparous young women. Association between thinness and age of marriage has been established by other researchers [29]. The pace of decline for thinness was lower among married adolescents (4% points) compared with young women (8%points). We have identified four states- Gujarat, Karnataka, Madhya Pradesh and Rajasthan- where prevalence of thinness and severe thinness was higher and persistent among married adolescents. Among these Gujarat and Rajasthan, along with Uttar Pradesh also had high prevalence of thinness among young married women.

Increase in overweight/obesity was similar, between 2005–06 and 2015–16 for both married nulliparous adolescents and married young women. The rapid increase in overweight or obesity is the mainstay of the nutrition transition that developing countries are undergoing [40]. Arunachal Pradesh, Punjab and Tamil Nadu have a higher prevalence of overweight or obesity and need to prioritize interventions for controlling and for treatment for overweight and obesity for both age groups.

This study also highlights several important findings with regard to possible determinants of malnutrition among married nulliparous adolescents and young women. Age, ethnicity, education and poverty influenced BMI among young women much more than among adolescents. Length of education mattered for improving BMI but not BAZ among married adolescents. At least 12 years of education was needed to improve BMI which is a concern for the 15 to 19 years aged married adolescent of which only 15% achieved this level of education. Belonging to tribal household can further aggravate nutritional vulnerabilities for both married adolescents and young women. Women belonging to tribal households have higher prevalence of thinness and limited access to maternal health services [41]. Belonging to poorest household reduced BMI of married young women more than adolescents. Adolescents and young women who wanted child later had a lower BMI than those wanting soon. Thus, there is an opportunity to reach women wanting child later with family planning services coupled with nutrition education and related services.

The results of this study indicate that a BMI cut-off close to 16.5 kg/m^2^ and 24.1 kg/m^2^ had a high sensitivity and specificity to screen thin and overweight or obese adolescents, respectively. Using current WHO BMI classification for adolescents overestimates prevalence of thinness and overweight or obesity. For adolescents, more research should be undertaken to determine valid BMI cut-offs for classification and other anthropometric techniques such as mid upper arm circumference (MUAC) explored. MUAC is known to be more predictive of acute undernutrition than BMI and has been endorsed by the government as a measure for pregnant women or lactating mothers aged 18 years and above [42,43].

Finally, the prevalence of severe thinness, though low, was persistent and comparable between adolescents and young women. Further, the findings highlight the need to reach to MARA or adolescents with double and triple burden of undernutrition (that is, those both short and thin or those short, thin and anemic) with a specialized package of services addressing thinness and anemia. Family planning should be an integral part of this package as delaying age at first birth in this age group can improve nutritional status, particularly impact child stunting as well as improve birth outcomes [28,44]. This small but most vulnerable group needs to be identified through community and facility based screening which includes measurement of height, weight and biochemical testing for anemia.

The results of this study have several programmatic and policy-related implications. First, newly married women should be actively brought under the ambit of health and nutrition programs and offered screening for assessing any nutrition risk (thinness, overweight/obesity, short stature, anemia). Such a service package has been developed for pregnant women and mothers and is being tested at several community and facility sites in India [45]. As mentioned in the introduction, platforms of reaching preconception nulliparous women are limited. The recently launched Anemia Mukt Bharat (Anemia free India) includes adolescent girls, newlywed and young married women (20–24 years) as target beneficiaries which could be tapped for reaching out with a larger package of nutritional interventions of supplementation, counselling and treatment if needed [26]. Community models that reach out to newlywed women or couples and link income generation to nutrition are also available for testing feasibility of scale-up [46].

The Government of India, under the National Institution for Transforming India (NITI) guidelines, prioritises 115 aspirational districts across the states to accelerate efforts in addressing anemia and undernutrition [47]. Among these districts, those with higher proportion of married adolescents and higher proportion of undernutrition in this age group should test delivery of a special service package addressing all nutritional risks among married adolescents and young women. Districts in the states of Gujarat, Karnataka, Madhya Pradesh and Rajasthan should be prioritized. Further, households belonging to scheduled tribes and in the poorest wealth quintiles should be prioritised in terms of reaching the most vulnerable. The one full meal scheme for pregnant women and lactating mothers in Andhra Pradesh, Karnataka and Telangana and the Mahatari Jatan scheme in Chhattisgarh provide tested strategies for providing special services to at nutrition risk pregnant women and lactating mothers, and could be extended to include newlywed women [48,49, 50]. Screening adolescents for overweight and obesity in schools and through special outreach camps biannually should be piloted in states like Arunachal Pradesh, Punjab and Tamil Nadu where prevalence of obesity in married young women is near or over 30%.

### Limitations

As this study uses cross-sectional survey, it cannot assess causality in the relationships of interest. Furthermore, the data are unable to account for the effects of seasonality on BMI of married nulliparous adolescents and married nulliparous young women. Weight for height is known to vary seasonally in young children [51, 52]. This paper also does not examine the effects of other variables such as, any illness prior to survey, decision making powers, experience of domestic violence and related empowerment indicators which may be related to both the independent variables of interest and the outcome [53]. Finally, the sample only includes married nulliparous women of reproductive age, with majority from rural settings. Findings may not be generalizable to the entire population of adolescent girls and young women. Examining the nutritional status of women prior to marriage represents an important area of future research that may have important programmatic implications to improving nutrition during the preconception period.

## Conclusion

Nutrition programs should identify the differential needs of thin, severely thin and overweight or obese married adolescents and young women. Coupling family planning and nutrition interventions is needed for both age-groups considering the lower BMI among adolescents and young women wanting child later. Adolescents and young women who face additional disadvantage because of their ethnicity, ability to access education or their level of poverty may require specific targeted interventions. Expanding platforms for pregnant and lactating mothers such as the community and facility based ante-and postnatal care services to include nulliparous women (newlyweds) should be tested in states with high burden of malnutrition among married nulliparous adolescent and married nulliparous young women. Screening tools for assessing nutrition status of married adolescents need to be further researched as using standard BMI based classifications overestimates thinness and overweight or obesity in this age group.

## Supporting information

S1 File Data from NFHS 3 (2005–06) and NFHS 4 (2015–16) used in this manuscript. (ZIP)
